# The ever-evolving world of microbes: the current state of microbial taxonomy, genome evolutionary dynamics, and the potential impact on the future of agricultural microbials risk assessment

**DOI:** 10.3389/fbioe.2025.1620652

**Published:** 2025-06-23

**Authors:** Katherine A. Karberg

**Affiliations:** Bayer Crop Science, Chesterfield, MO, United States

**Keywords:** agricultural biologicals, microbial genome evolution, horitzontal gene trasfer, microbial taxonomy, pangenome, regulation, risk assessment, biosafety

## Abstract

Risk assessment frameworks for plant agricultural biotechnology products have been in place for decades, focused on the evaluation of living biotechnology products created through genetic engineering. These products contain genetic material from outside the breeder’s gene pool, which is often from different taxa or represents “novel combinations of genetic material”. These products are typically considered to be “genetically modified” (GM) organisms in regulatory jurisdictions. However, in the microbial world, particularly among Bacteria and Archaea, the rapid expansion of genome sequence databases shows that natural microbial innovation primarily occurs through the natural exchange of genetic material from various sources, even from different taxa. This means that many microbes can be considered naturally occurring GM organisms. This raises the question of whether labeling a microbe as GM is always scientifically relevant for risk assessment. In most regulatory frameworks, being classified as GM significantly impacts the registration path, especially for microbes intended for environmental release. A more effective and science-based regulatory approach would assess the actual functions of a microbe rather than relying on the uncertain classification of its genetic material. This would benefit regulators, developers, and society by promoting the use of microbial technologies for agricultural use.

## 1 Introduction

Microbial biologicals have been used in agriculture since the turn of the 20th century; however, challenges related to consistent efficacy, stability, production scalability, and other obstacles have prevented them from becoming a primary tool in agricultural production (Batista and Singh, 2021; Debnath et al., 2020). Advances in basic microbiology knowledge and production capabilities, the need for novel and complementary approaches in modern crop production, and societal drivers on sustainability collectively create an opportunity for the use of microbial biologicals. A limiting factor for realizing the potential of microbial biologicals is fit-for-purpose regulatory systems specific to the biological realities of microbes; especially for microbes with intentional genetic modifications, including the transfer and integration of genes from different taxa (EFSA Scientific Committee et al., 2020; Kerr and Bullard, 2020; Thakor and Charles, 2025).

Biotechnology regulatory systems have been in place and effective for plant biotechnology innovations since the mid-1980s (Gleim and Smyth, 2018). However, these systems are predicated on the idea that transfers of genes from outside the breeder’s gene pool leading to “novel combinations of genetic material” (NCGM), particularly between different taxa, would not be naturally derived due to presumed natural barriers of gene transfer. This leads to the belief that such transfers represent a violation of “natural laws” and carry inherently greater risk. This plant biotechnology framework has thus far carried over to microbial biologicals. Accordingly, current risk assessment paradigms for microbial products with added gene content or other changes that categorize them as GM or NCGM are sometimes more intensive than those deemed as “conventional” or “wild type”. This can present a financial barrier and significant increase in timelines to development, may restrict large scale field trials that are essential to demonstrate efficacy, and potentially rule out effective microbial solutions for farmers (Chemla et al., 2025).

In the time that global biotechnology risk assessment frameworks have been in place, the genomic era led to a rapid expansion of the sequence database across the tree of life which has increased the knowledge of microbial diversity and evolution. The analyses of bacterial and archaeal genomes have changed the “conceptual foundations of microbiology” (Koonin et al., 2021). Rather than fixed entities, most bacterial and archaeal genomes are now understood to be dynamic, with constant genetic flux (Arnold et al., 2022; Brito, 2021; Gophna and Altman-Price, 2022). Horizontal gene transfer (HGT), even between distantly related taxa, is the dominant mechanism of genetic innovation (Dmitrijeva et al., 2024; Sheinman et al., 2021). However, HGT dynamics remain an active area of investigation (Arnold et al., 2022; Brito, 2021). The abundance of microbial sequence data has also changed microbial taxonomic practices (Hug, 2024; Hugenholtz et al., 2021).

Many features of microbial evolution do not easily reconcile with current risk assessment frameworks that depend heavily on whether a microbe is designated as GM with genes acquired from different taxa or NCGM. The example of the *Paenibacillus* genus illustrates this point with its expansive genetic diversity and ever-evolving taxonomy. *Paenibacillus* is a diverse bacterial lineage long noted as a potential treasure trove for biotechnology uses in agriculture, human and veterinary medicine, bioremediation, and other industrial uses (Grady et al., 2016). This includes the use of whole *Paenibacillus* strains, known as microbial biologicals, as plant growth promoting bacteria in agriculture (Padda et al., 2017).

### 1.1 Microbial taxonomic practices are actively evolving

Taxonomic classification of microbes is an important, but evolving discipline (Hackmann, 2025; Hugenholtz et al., 2021; Oren et al., 2023; Riesco and Trujillo, 2024). The abundance of microbial sequence data has changed microbial taxonomic practices, leading to debates on consensus methods and the biological relevance of taxonomic ranks, including the longstanding debate over how to define a microbial species (Vernikos et al., 2015). The ongoing evolution of microbial taxonomy is critical for understanding the complexities of microbial life. However, it also directly impacts risk assessment frameworks, regulatory practices, and the effective application of microbial biologicals in agriculture and environmental management.

There are a variety of historical and current approaches for assigning microbial taxonomic ranks, but even those that propose consensus standards are often soon challenged by new research (Hackmann, 2025; Hugenholtz et al., 2021; Parks et al., 2018; Riesco and Trujillo, 2024). Initially, microbial taxonomy was based on phenotypic characterizations such as morphology, biochemical testing, lifestyle (e.g., pathogenic), and habitat (Hugenholtz et al., 2021). However, phenotypic approaches are limiting for elucidating evolutionary relationships, as distantly related microbes can have shared features, such as coexisting in a habitat or sharing metabolic capabilities such as nitrogen fixation.

The ability to compare genetic sequences was a critical paradigm shift for microbial taxonomy. DNA:DNA hybridization was one of the first methods to approximate genetic relatedness, followed by more comprehensive gene sequencing (Brenner, 1973). The discovery of the 16S rRNA gene as a slowly evolving, universally present non-eukaryotic microbial gene was a major step forward in microbial molecular phylogeny and led to the reframing of the tree of life into three different domains, including the discovery of the Archaea as a domain distinct from Bacteria and Eukarya (Woese and Fox, 1977). In the genomic era, taxonomic classifications now rely heavily on multi-sequence genome-based classifications, using metrics such as average nucleotide identity (ANI) to delineate species, supplemented by phenotypic information (Parks et al., 2020; Riesco and Trujillo, 2024).

However, at least 85% of microbial life is estimated to be unculturable and thus has no phenotypic information (Rinke et al., 2013). Sequence-based classifications allow for phylogenetic and taxonomic explorations into the uncultured realm, derived from metagenome-assembled genomes and single cell genomics. However, even if taxonomic ranks can be assigned based on sequence thresholds, there is no consensus on how to name such species according to the International Code of Nomenclature of Prokaryotes, as there is no representative physical sample to archive (Hugenholtz et al., 2021; Oren et al., 2023). That is, for most microbial diversity, there is no reference strain, no “wild type” or “conventional” counterpart, let alone consensus nomenclature for formally naming such species. As more genome sequences are added, a taxonomic system for uncultured microbes is resolved, and phylogenetic methods advance, further revisions of the tree of life are anticipated (Eme and Tamarit, 2024; Hug, 2024).

### 1.2 The microbial pangenome concept and its use in taxonomic classifications

Modern sequence-based taxonomic methods rely on sequence attributes that are held in common between organisms. However, there is significant genetic diversity even at the species level for many microbes. For example, *Escherichia coli* strains exhibit vastly different lifestyles, ranging from non-pathogenic lab, commensal, and environmental strains, as well as different pathogenic strains that cause significant human diseases. These variations arise from their different genetic content. For example, pathogenic *E. coli* strains have on the order of 300-1,000 more genes than the non-pathogenic ones (Rasko et al., 2008).

Extensive species-level genetic diversity permeates bacterial and archaeal lineages, leading to the pangenome concept (Medini et al., 2005). The total collection of genes in a lineage is the pangenome. The genes held in common are the core genome, typically representing operational genes for basic cell functions including DNA replication, transcription, and translation machinery. The remaining genetic diversity not held in common is the accessory (or variable, dispensable, auxiliary, amongst other names) genome. Accessory genes generally reflect functional adaptations to specific lifestyles, such as metabolic clusters, virulence factors, defense mechanisms, and more (Sheinman et al., 2021).

Core genes, due to their universal presence, are presumed to be vertically inherited making them reliable to construct phylogenies for taxonomic ranking (Hugenholtz et al., 2021; Parks et al., 2018). Yet, there is debate that using core genes are not sufficiently representative to define taxonomic ranks, because so much of the representative genetic diversity in a lineage, the accessory genome, is excluded, potentially missing additional cohesive forces that shape microbial genomes (Douglas and Shapiro, 2024; Zhu et al., 2019).

### 1.3 The dynamic nature of microbial accessory genes

The ever-increasing amount of sequence data reveals that for many microbial lineages the accessory genome contains more genes than the core genome, in the most extreme cases representing 80% of the lineage gene content (Tettelin and Medini, 2020). The current sequence database is not yet saturated for many microbial lineages, meaning that the gene pool at a given taxonomic designation continues to grow, expanding the accessory genome (Lapierre and Gogarten, 2009). This is referred to as an open pangenome.

Accessory genes are thought to often be acquired through HGT processes, rather than vertically inherited (Dmitrijeva et al., 2024). These genes often exhibit sequence characteristics (G + C content, codon bias) that are distinct from core genes. They may also be linked to chromosomal markers that indicate gene transfer, such as insertion sequence elements and integrated phage elements, or reside on extrachromosomal mobile elements such as plasmids, which facilitate their movement between different taxa. However, bacterial and archaeal genomes do not grow indefinitely; there is evidence that accessory genes are also often lost over time. Additionally, these genes can undergo adaptive mutations when they are integrated into new host organisms. As a result, accessory genes are particularly dynamic compared to core genes.

The evolutionary dynamics of HGT and the accessory genome are not fully understood, even though they make up a significant portion of the genes in many microbial lineages (Domingo-Sananes and McInerney, 2021). It is thought that genes transferred between closely related organisms are more likely to evade the host’s defenses against “non-native” DNA, such as restriction-modification systems and CRISPR-Cas9. These closely related genes may also integrate more easily into the recipient’s genome if they share similar features and functions. However, gene sharing is not limited to closely related organisms; it also occurs between distantly related lineages across taxonomic ranks, including examples of HGT between different domains of life, especially Bacteria and Archaea (Gophna and Altman-Price, 2022; Kloesges et al., 2011). Further, for some lineages, codon bias analyses of the accessory genome, and especially the most recently horizontally acquired genes, demonstrate that they are more similar to each other in codon bias than the core genes are, suggesting that they may be from a common gene pool that is shared beyond the genus level (Karberg et al., 2011). What, if any, implications a gene pool that extends across taxonomic ranks has on taxonomy remains to be determined. Regardless, much is still to be discovered about the dynamic nature of microbial genomes and what the vast genetic diversity in a lineage means for phylogeny and taxonomic designations.

### 1.4 The *Paenibacillus* genus illustrates the evolving nature of microbial genomes and taxonomy

The *Paenibacillus* genus, of great interest from a biotechnology perspective due to its diverse functional content, exemplifies the uncertainties in microbial taxonomy and genome evolution. Taxonomic revision within the *Paenibacillus* genus is common and ongoing (Grady et al., 2016). For nearly 100 years, strains were classified as members of the *Bacillus* genus based on basic phenotypic characteristics such as morphology, oxygen respiration, and endospore formation. However, more advanced biochemical and phenotypic analyses conducted in the late 1980s indicated the presence of distinct groups within these strains. In 1993, 16S rRNA gene sequencing confirmed the distinct groups, each proposed as novel genera, including the *Paenibacillus* genus (Grady et al., 2016). *P. polymyxa* was designated at the genus type strain in 1994, not because of its evolutionary or biological significance for the genus, but due to the historical circumstance of being one of the better characterized *Paenibacillus* strains at the time, having been cultured in 1880 (Pandey et al., 2023).

In the genomic era, the genetic diversity of *Paenibacillus* genus continues to expand. It is already expected that the genus will be reclassified into novel genera (Grady et al., 2016). There are also ongoing proposals for novel species, including a recent study of *P. polymyxa* strains supporting that the lineage be split into four different species based on genome sequence metrics, coupled with phenotypic data (Maggi et al., 2024). In this same study, a pangenome analysis revealed that even if the current strains are reclassified into four new species, each new species has an open pangenome. That is, the extent of the genetic diversity is not at saturation even at the species level of the type strain and the gene pool of *Paenibacillus* at any taxonomic rank cannot yet be defined.

### 1.5 The *nif* regions in the *Paenibacillus* genus are part of the accessory genome, are non-native, of variable organization, and are actively undergoing evolution in natural strains

A key focus of agricultural microbials is developing microbes to fix nitrogen in crops to reduce the need for synthetic fertilizers. Nitrogen fixation is energetically expensive, and microbes repress this pathway if sufficient environmental nitrogen is available (Fan et al., 2019). Even if microbes naturally possess such capabilities and have a symbiotic relationship with plants (legumes), they may not work efficiently in agricultural settings where synthetic nitrogen is already used. Precise engineering of this pathway in microbes has the potential to upregulate biological nitrogen fixation in typical agricultural settings, thus reducing the need for synthetic fertilizers (Wen et al., 2021).

Biological nitrogen fixation genes, including the *nif* genes, are part of the accessory (Xie et al., 2014). N-fixing genes in this lineage have variable organization, indicating that they are prone to significant evolutionary change. Nitrogen fixation is not an ancestral trait of *Paenibacillus*; rather it has been acquired through multiple HGT events from phylogenetically distant taxa. The donor for at least one of the *nif* HGT events is from *Frankia*, which belongs to a different bacterial phylum, Actinomycetota. Another donor of the nitrogen fixation genes appears to be from another domain of life, a methanogenic archaeon. This aligns with the evolving understanding of HGT, where gene transfer potential is influenced by the total gene pool in an ecological niche, even occurring between distantly related members (Arnold et al., 2022; Dmitrijeva et al., 2024).

The differential organization of the *nif* genes in *Paenibacillus* strains supports that evolution of the region is ongoing. The genes are organized into at least two different subgroups, with variation in gene organization and structure even within subgroups (Xie et al., 2014). This differential organization is the result of horizontal acquisition, gene loss (including total loss of the region in some strains), reacquisition of some *nif* and other functionally related genes, and additional sequence evolution, including changes in promoter regions.

## 2 Discussion

Advancing agricultural microbials requires regulatory systems with fit-for-purpose risk assessments that allow large-scale field trials to demonstrate efficacy. Currently, most global regulatory systems do not provide efficient pathways, if they exist at all, for commercialization of intentionally modified microbes, including those with genetic elements from different taxa or NCGM. Given the understanding that HGT is the main mechanism of microbial innovation, that gene pools and NCGM are not yet circumscribed because microbial sequence diversity is far from saturation, and the ongoing evolution of microbial taxonomy, it is necessary to reevaluate risk assessment frameworks based on these criteria.

HGT occurs across taxonomic ranks, including between different domains of life. Most microbes constantly sample and obtain genes from external sources, both cellular and acellular, then integrate, mutate, and lose them, using their own “genetic engineering” methods, similar to those used for intentional modifications. The scientific basis of risk assessment distinguishing acquired genetic elements by donor taxa especially must be revisited. A nitrogen fixation gene transferred between different bacterial phyla is not expected to have a different hazard profile than an intrageneric one, nor is it violation of “natural laws” if this is a phenomenon already found in nature.

Microbial taxonomy is also apt to change as the genome databases grow, the methodologies for comparing genomic information and deriving phylogenies advances, along with the knowledgebase of microbial evolutionary dynamics. The reality today is that the debate on a biologically relevant consensus definition of a bacterial or archaeal species is ongoing. In a regulatory context, if a gene is initially considered intrageneric is reclassified as intergeneric, does that change its hazard or the ethical ramifications of sourcing it?

For many microbial lineages, like *Paenibacillus*, the question of whether an engineered strain represents NCGM (including genes from other taxa) cannot accurately be answered, because the pangenome is open and the sequence databases are not at saturation. This means that the gene pool of the *Paenibacillus* genus is not yet circumscribed. The patchwork presence of nitrogen fixation pathway in some *Paenibacillus* strains illustrates several characteristics of the accessory pangenome. These genes are not fixed, and they experience gene gain from distant taxa, gene loss, and mutation to adapt to specific ecological roles. There is no definitive “wild type” or “conventional” version of the genes or pathway, and the availability of additional genome sequences continues to reveal NCGM. These concepts apply to other taxa that are of great interest as agricultural microbials, including *Bradyrhizobium* (Terra et al., 2025; Zhong et al., 2024).

Establishing a framework that ensures intentionally modified microbes can be safely used in agriculture is paramount. Furthermore, an objective, standardized safety evaluation of the final product should be the critical criteria, rather than focusing on the processes by which they were developed or on criteria that are subject to change. Microbes can be tested by a variety of methods, and potential strategies for biocontainment and reduction of other risks can be implemented, such as those recently described by Chemla et al. (Chemla et al., 2025). However, the testing should be fit-for-purpose for the intended use. For example, a non-animal-pathogenic microbe with modifications to the nitrogen fixation pathway would not be expected to have enhanced human pathogenicity, so a fit-for-purpose risk assessment may not require an extensive animal testing package; if information on animal pathogenicity were deemed necessary, safety information (in the form of literature searches on pathogenicity or lack thereof, or even preexisting animal data) can be bridged from a related strain.

Progress is ongoing in both the development of regulatory frameworks for agricultural biologicals and in the knowledge of microbial genetic diversity, genome evolutionary dynamics, and taxonomy. Even amidst new discoveries in the microbial world, the potential of innovative agricultural biologicals to address environmental and societal challenges can be realized if regulatory frameworks are anchored in objective criteria and testing methods.
